# Editorial comment: Pre-eclampsia

**DOI:** 10.1093/emph/eoy030

**Published:** 2018-10-13

**Authors:** Gillian R Bentley

**Affiliations:** Department of Anthropology, Durham University, Durham, UK

Pre-eclampsia (PE), marked symptomatically by increased blood pressure, renal or liver insufficiency, cerebral disturbances, excessive protein in urine (proteinuria) or low platelet levels and edema is a potentially fatal condition that affects a small proportion (estimated at 4.6%) of women around the globe [1]. Risk for women is increased by twin or higher gestations, age, primipaternity, an elevated body mass index, metabolic disorders and smoking among other factors [1]. The pathophysiology of this disease is otherwise not well understood; treatment is limited to alleviating symptoms and, in more severe cases, inducing labour or performing pre-term C-sections [2]. Maternal mortality associated with the condition is low relative to deaths experienced during labour and delivery, but presumably higher in regions where good medical facilities are absent. Due to the relatively small numbers of women who experience PE, it would be rarely documented among populations such as hunter-gatherers where lifestyles are presumably closer to those in which our species evolved. Apes (with among the most highly invasive trophoblasts) are the only species thought to experience this condition [3].

Evolutionary perspectives on this condition emerged with David Haig’s publication on parental genetic conflicts [4]. He argued that paternally derived genes will attempt to mediate additional resources to aid foetal growth, while maternally derived genes will attempt to control these resources in the interest of future reproductive effort. It is this conflict that, allegedly, leads to PE in mid-to-late pregnancy. Clinical studies of PE generally do not contextualize its potential evolutionary origin, but focus instead on potential proximate causes for the condition such as twin or higher gestations, age, smoking and metabolic disorders, although some sources acknowledge risk factors such as primipaternity which may also have an adaptive function.

Clinical Briefs in EMPH provide short reviews of evolutionary approaches to pathologies or conditions in order to broaden the scope of thinking in medicine and public health, and that could help in finding novel treatments. Serendipitiously, two complementary Clinical Briefs on the topic of PE were simultaneously submitted to EMPH by separate sets of authors [5, 6]; we are publishing them together and briefly highlight specific contrasts.

Although both focus on the genetic conflict model described above as well as immunological dysfunctions known to increase risk for PE, Varas Enriquez *et al.* (2018) also discuss how the nutritional requirements of large-brained infants may have exacerbated genetic conflicts during gestation. They draw attention to the suggestion that low rates of human fecundability function to increase sexual exposure to partners prior to conception, thereby lowering PE risk.

Buschman *et al.* (2018) are pessimistic that clinicians could effectively provide a cure given the suggested underpinnings of PE in genetic conflict. They do speculate, however, that future epigenetic studies may be able to identify the effects of paternally derived genes that could be targeted for interventions. They also argue that understanding the evolutionary roots of PE may enable better prediction of who is vulnerable among pregnant women. Similarly, Varas Enriquez et al. suggest that providing evolutionary explanations to patients about PE’s origin could be psychologically beneficial. These authors also argue that further studies of placental evolution would benefit our understanding of PE. In sum, both briefs contextualize PE within a broader, deeper theoretical framework in order to shed further light on this complex condition. 

**Conflict of interest**: None declared.
